# The Potential of Phytomelatonin as a Nutraceutical

**DOI:** 10.3390/molecules23010238

**Published:** 2018-01-22

**Authors:** Marino B. Arnao, Josefa Hernández-Ruiz

**Affiliations:** Department of Plant Biology (Plant Physiology), Faculty of Biology, University of Murcia, 30100 Murcia, Spain; jhruiz@um.es

**Keywords:** antioxidant, cancer, circadian rhythm, dietary/food supplements, free radicals, fruits, medicinal plants, melatonin, neurological diseases, nutraceutical, phytomelatonin, plant foodstuffs, sleep disorders

## Abstract

Phytomelatonin (plant melatonin) is chemically related to the amino acid tryptophan and has many diverse properties. Phytomelatonin is an interesting compound due to its outstanding actions at the cellular and physiological level, especially its protective effect in plants exposed to diverse stress situations, while its vegetable origin offers many opportunities because it is a natural compound. We present an overview of its origin, its action in plants in general (particularly in plant species with high levels of phytomelatonin), and its possibilities for use as a nutraceutical with particular attention paid to the beneficial effects that it may have in human health. The differences between synthetic melatonin and phytomelatonin, according to its origin and purity, are presented. Finally, the current market for phytomelatonin and its limits and potentials are discussed.

## 1. Introduction

Melatonin (*N-*acetyl-5-methoxytryptamine) is a ubiquitous molecule in nature, with biological activities in unicellular organisms, fungi, plants, and animals. Melatonin is broadly known as a biological modulator of circadian rhythms, mood, sleep, body temperature, locomotor activity, food intake, retinal physiology, sexual behavior, and the immune system [1,2,3,4,5,6,7]. Moreover, melatonin is an excellent antioxidant at physiological concentrations [8,9,10]. Also, many cancer types have been associated with low melatonin levels in the bloodstream, resulting in melatonin being used as a treatment to stop or decelerate the growth of cancerous cells [11,12,13,14,15].

In the following section, we present an overview of the most relevant aspects of phytomelatonin, including its origin and discovery in plants and its physiological roles therein, focusing on its most novel aspects as a nutraceutical compound and the beneficial effects that it may have in healthcare. The differences between synthetic melatonin and phytomelatonin are presented. Also, the current market for phytomelatonin and its limits and potentials are discussed.

## 2. Phytomelatonin in Plants

**Figure 1:**
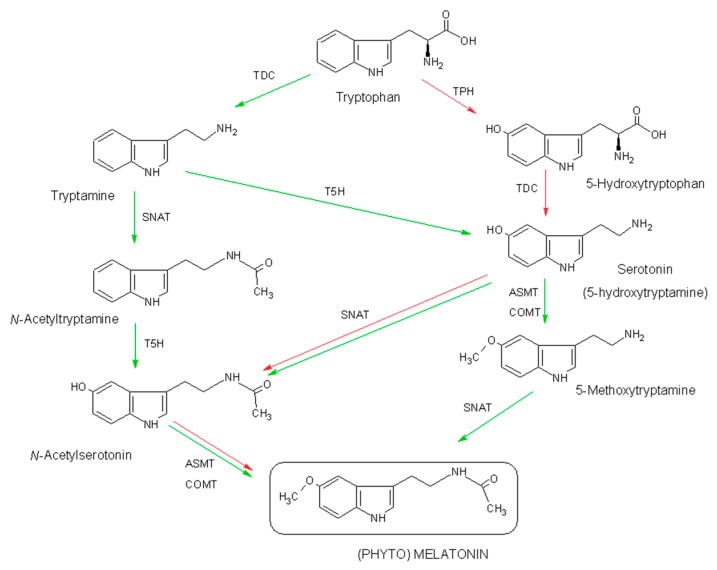
Biosynthetic pathways of phytomelatonin. The enzymes of the respective steps are: T5H—tryptophan 5-hydroxylase; TDC—tryptophan decarboxylase; TPH—tryptophan hydroxylase; SNAT—serotonin *N*-acetyltransferase; ASMT—acetylserotonin methyltransferase, and COMT—caffeic acid *O*-methyltransferase. The green and red lines show the predominant pathway in plants and in animals, respectively.

The molecular structure of phytomelatonin (*N-*acetyl-5-methoxytryptamine) is an indoleamine derivative of the amino acid tryptophan (Figure 1). While melatonin is the term used to name to the compound of animal origin or obtained by chemical synthesis, the term phytomelatonin refers to melatonin of plant origin. The term phytomelatonin was first proposed in 2004 in a paper about the treatment of liver cancer in rats. In this article, according to the authors, “*… phytomelatonin could have a potentially significant impact as a new strategy in human cancer prevention*” [16].

The first description of endogenous phytomelatonin in higher plants was provided in 1993 by van Tassel and co-workers in a congress communication [17]. The authors described how they identified phytomelatonin by radioimmunoassay (RIA) and gas chromatography by mass spectrometry (GC-MS) in the ivy morning glory (*Pharbitis nil* L., synonym *Ipomoea nil* L.) and in tomato fruits (*Solanum lycopersicum* L.); the results were published extensively in 1995 [18]. Also in 1995, authors of two publications demonstrated the presence of phytomelatonin in higher plants. Dubbels and co-workers used RIA and HPLC-MS to measure the phytomelatonin levels in extracts of *Nicotiana tabacum* L. and in five edible plants [19]. Two months later, the presence of phytomelatonin in a large number of edible plants was quantified by RIA and liquid chromatography (HPLC) with fluorescence detection by a Japanese group [20]. Another communication appeared in the same year, in which a Czech research group identified the presence of melatonin in *Chenopodium rubrum* L. using liquid chromatography with mass identification (LC-MS/MS) [21]. Successive studies have quantified phytomelatonin in many plants and it is now accepted that phytomelatonin is present in all plants, although there are no data available for non-vascular plants, probably because they have not yet been studied.

### 2.1. Biosynthesis of Phytomelatonin

Melatonin and phytomelatonin are synthesized from the amino acid tryptophan in pathways that have been extensively studied in both animals and plants [22,23,24]. In plants, tryptophan is converted into tryptamine by tryptophan decarboxylase (TDC) (Figure 1). Tryptamine is then converted into 5-hydroxytryptamine (commonly known as serotonin) by tryptamine 5-hydroxylase (T5H). *N*-acetylation of serotonin is catalyzed by the enzyme serotonin *N*-acetyltransferase (SNAT). *N*-acetylserotonin is methylated by acetylserotonin methyl transferase (ASMT), a hydroxyindole-*O*-methyltransferase, which generates (phyto)melatonin. In plants, methylation of *N*-acetylserotonin can also be carried out by caffeic acid *O*-methyltransferase (COMT), a class of plant enzymes that can act on a variety of substrates, including caffeic acid and quercetin [25]. Serotonin may also be transformed into 5-methoxytryptamine by ASMT and by COMT to generate phytomelatonin through the action of SNAT. Additionally, as a possibility phytomelatonin could be generated by formation of *N*-acetyltryptamine, which is converted into *N*-acetylserotonin, according some data of *Sekiguchi* mutant rice [26,27]. Finally, serotonin can also be formed from 5-hydroxytryptophan after the action of tryptophan hydroxylase (TPH) and TDC, the latter step occurring mainly in animals but also, to a lesser extent, in plants. Moreover, phytomelatonin can be generated through the formation of 5-methoxytryptamine, as proposed by several authors, suggesting that the phytomelatonin biosynthesis pathway may follow more alternative routes compared with the one in animals, reflecting a greater capacity to adapt to metabolic changes [28,29]. Thus, (phyto)melatonin can be synthesized in many ways, the most relevant being the sequence: tryptophan → tryptamine → serotonin → *N*-acetylserotonin → phytomelatonin (Figure 1). All above enzymes have been detected and characterized in rice and *Arabidopsis*, except TPH, which is well-known in animals but not in plants. In animals, the main route is: tryptophan → 5-hydroxytryptophan → serotonin → *N*-acetylserotonin → melatonin (Figure 1). Melatonin intermediates are produced in various subcellular compartments, such as the cytoplasm, the endoplasmic reticulum, and chloroplasts or mitochondria [23]. Besides this, melatonin in plants is not a final product in the route since it is usually hydroxylated, forming 2-, 3-, and 6-hydroxy-melatonin as products, with 2-hydroxymelatonin the major metabolite [30].

### 2.2. Levels of Phytomelatonin

Since the discovery of phytomelatonin in plants, a number of studies have reported on its detection in a variety of vegetables, fruits, seeds and medicinal herbs, and also in wild plants [31,32,33]. Table 1 shows some of the edible plants identified as having a high phytomelatonin content. In general, any attempt at classification according to the phytomelatonin content is very difficult and so the data are highly variable, varying from a few picograms up to micrograms per gram of material analyzed. This is because the determinations of phytomelatonin were not very accurate in the first years, mainly due to methodological problems, and also because there has been little control of the environmental conditions of cultivation and conservation of the plant material (see below). Values of phytomelatonin content are shown in Table 1, which depicts the data published so far on edible plant species with phytomelatonin content above 10 ng/g tissue. To date, the phytomelatonin content of coffee beans is the highest recorded in plant material. Roasted coffee beans showed an even higher phytomelatonin content than green beans, reaching 60–78 ng of phytomelatonin per mL of brewed coffee [34]. Taking the maximum levels observed for each species, apple and cherry also had a high content. Of the *Solanaceae* family, besides Goji berry, tomato and pepper fruits showed a high phytomelatonin content. Of note is the high dispersion of data obtained for the several varieties of *Vitis vinifera* that have been analyzed. In addition, mustard seeds showed high phytomelatonin levels.

Table 2 shows the data of some aromatic/medicinal plant species with phytomelatonin contents above 100 ng/g tissue. As can be seen, the phytomelatonin content of thymus, sage, Chinese liquorice root, peppermint and St. John’s wort showed contents above 19 µg/g dry weight (DW). Also, aloe, clove, yarrow, and figwort showed phytomelatonin contents that were much higher than those recorded for most of the edible plants listed in Table 1. In general, aromatic and medicinal plants had higher levels of phytomelatonin than seeds and fleshy fruits, while leaves, stems, seedlings and roots presented higher phytomelatonin content than fruits (data not shown).

The ripeness stage and post-harvest conservation are important factors in the phytomelatonin content of plant tissues, and are decisive in the case of fruits. The influence of cultivation conditions and environmental factors on phytomelatonin content is a determining factor. Temperature, soil humidity, solar radiation (visible and UV), and soil chemicals play a relevant role in phytomelatonin content in plants. Plants cultivated in field conditions tend to show a higher phytomelatonin content than plants cultivated in growth chambers, where light, temperature and humidity are controlled. For example, tomato plants cultivated in field conditions have up to 3 times the phytomelatonin level of those grown in culture chambers [49]. In general, the phytomelatonin content in plants increases in stress situations, protecting and strengthening the plants through an anti-stress response. Biotic stressors such as infection by bacteria, fungi, viruses, and mechanical stressors such as those induced by herbivores or insects provoke high phytomelatonin biosynthesis in plant tissues as a defense response [28,50,51].

**Figure 2:**
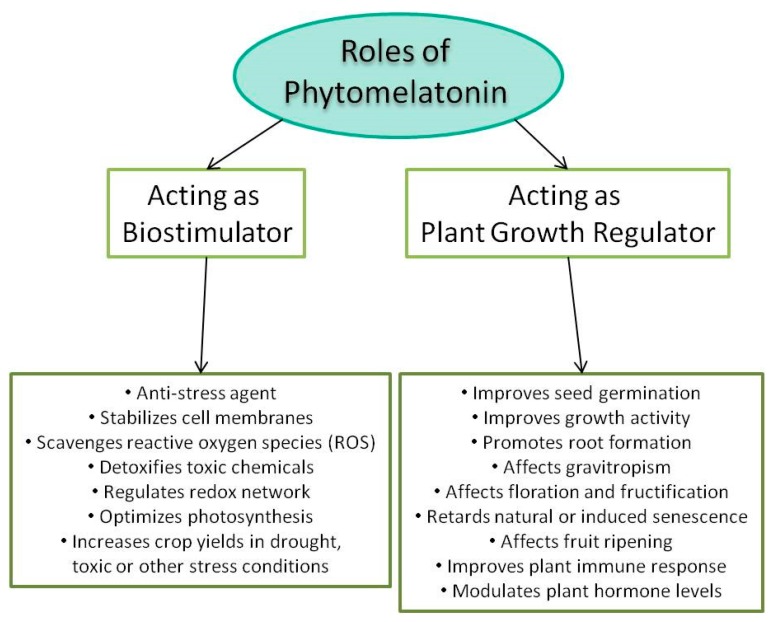
Studied roles of phytomelatonin in plant physiology.

With regard to the role of phytomelatonin in plants, although it was initially thought that its possible role would be closely related to the one known in animals, a fresh wave of studies in plants confirmed that it plays multiple roles in the physiology of plants. Although the details of these papers are beyond the scope of this review, Figure 2 summarizes the roles of phytomelatonin studied so far in plants. Note that there are excellent reviews on the subject [28,32,50,51,52].

## 3. Phytomelatonin as a Nutraceutical

The word nutraceutical, made from the terms “nutrient” and “pharmaceutical”, was coined by Stephen DeFelice, who defined nutraceuticals as “foods (or part of a food) that provide medical or health benefits, including the prevention and/or treatment of a disease” [53]. Currently, the term “nutraceutical” applies to a wide range of products, such as dietary supplements, herbal/botanical products, specific processed foods (functional foods) and also isolated nutrients. The European Nutraceutical Association (ENA) defines nutraceuticals as substances that differ from pharmaceuticals, which are “synthetic substances or chemical compounds formulated for specific indications”. The terms “dietary supplements” and “functional foods” are used without distinction as synonyms, although there are substantial differences between them, which are not always evident. Thus, dietary supplements contain nutrients derived from food products and are commonly concentrated in capsule, powder, liquid or tablet forms; functional foods contain the nutrients necessary for survival; and nutraceuticals are complementary to the diet, but also aid in the prevention of diseases and health disorders [54].

There are numerous classifications of nutraceuticals, functional foods, and dietary supplements. They were previously categorized as either potential or established nutraceuticals on the basis of the food material and nutrients or in terms of their effects on the body. Most commonly, their classification is based on the chemical constituents or active ingredients. In recent years, many new nutraceuticals have been added, forming a long list whose active ingredients are very diverse and sometimes strange, if not surprising. They include phenolic compounds (i.e., flavonoids, anthocyanins, resveratrol), organic acids (vitamin C), tocopherols (vitamin E), carotenoids (provitamin A), anthraquinones, terpenes, alkaloids, isothiocyanates, and mono- and poly- unsaturated fatty acids (MUFAs, PUFAs), among others. A particular type refers to pre- and pro-biotic products. Consumers in the USA, Canada, Europe and Japan demonstrate great acceptance of these products, and readily pay the premium prices that the most of them have. For nutraceutical producing companies, the potential nutritional interest is perhaps outshone by the prospective market value that is expected to reach the US$250 billion mark by 2018 [55,56,57,58,59].

Generally, nutraceuticals of plant origin (plant-derived foods) tend to be more accepted by consumers than others. In this sense, nutrition-based healthcare during the history of mankind can be explained by the humorous commentary of S.B. Rowe [60]:

2000 B.C.—Here, eat this root.
1000 A.D.—That root is heathen. Here, say this prayer.
1850 A.D.—That prayer is superstition. Here, drink this potion.
1940 A.D.—That potion is snake oil. Here, swallow this pill.
1985 A.D.—That pill is ineffective. Here, take this antibiotic.
2000 A.D.—That antibiotic is artificial. Here, eat this root.

### 3.1. Melatonin in Humans

**Figure 3:**
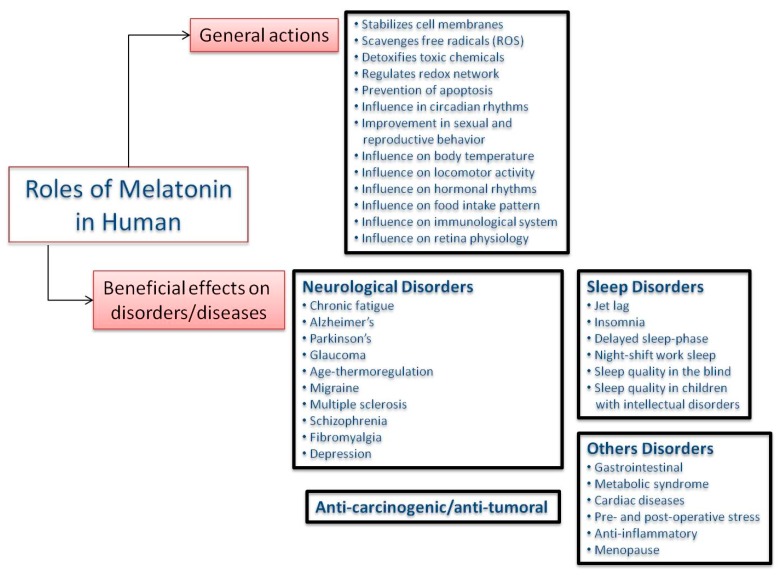
Roles of melatonin in human physiology.

Taking into account the wide range of activities of melatonin in human physiology, where it acts as a modulator of several processes such as mood, sleep, body temperature, locomotor activity, food intake patterns, circadian rhythms, and immunological regulation, among others (Figure 3), the ingestion of melatonin (synthetic or phytomelatonin) can be expected to play a role in some important aspects of human physiology, leading to effects on diseases and disorders. Indeed, melatonin is widely used for therapeutic purposes. To date, the use of melatonin has been restricted to the improvement of sleep quality, the alleviation of feelings of jet lag, and the reduction of sleep onset latency. However, numerous studies have concluded that melatonin could also be associated with the prevention of several diseases related to ageing and oxidative stress, including type 2 diabetes, cardiovascular and neurodegenerative diseases or cancer [2,3,4,5,11,12,15,61,62,63,64] (see Figure 3).

The intake of ‘natural products’ in a balanced diet is considered a positive contribution to human health. While nutritional supplements are many and varied, dietitians and nutritionists are increasingly recommending the consumption of natural foods with proven health-promoting effects, such as fruits, vegetables, cereals, nuts, vegetable oils, and beneficial herbs, among others. Thus, the intake of phytomelatonin could be correlated with the consumption of plant foodstuffs. Phytomelatonin from plants is absorbed from the gastrointestinal tract, modulating blood melatonin levels. Melatonin has a half-life of about 20–40 min in blood, after which its levels decay as a result of metabolism and elimination in urine [65,66,67].

The scientific literature provides several examples of the effect that phytomelatonin absorption has or could have on human health. In rats fed walnuts [68] and in chicks fed grains [20], an increase in serum circulating melatonin can be observed. In these cases, the intake of phytomelatonin-rich plants produces an up to 4-fold increase in basal melatonin levels in the bloodstream. This increase occurs 60–120 min after the consumption of phytomelatonin-rich plants. In the case of rats fed walnuts, a correlated increase in antioxidant activity in blood was observed, indicating that phytomelatonin (and also other phyto-substances) improve the antioxidant pool. In another study in rats, the intake of germinated kidney beans (*Phaseolus vulgaris* L.) altered the melatonin and serotonin levels in plasma and 6-sulfatoxymelatonin (the main urinary degradation metabolite of melatonin in humans) in urine. Moreover, melatonin plasma bioavailability derived from kidney bean sprouts was compared with synthetic melatonin intake. The kidney bean sprout extract was characterized as containing 529 ng/g of phytomelatonin. Ninety minutes after the intake of kidney bean sprout extract, plasmatic melatonin and urine 6-sulfatoxymelatonin levels had increased by 16%, suggesting that kidney bean sprouts could be a good source of dietary phytomelatonin [37].

Similar effects have been found in humans. Thus, the consumption of sweet cherries (prepared as a powdered freeze-dried product) led to a rise of 6-sulfatoxymelatonin in young, middle-aged, and elderly subjects. Generally, a direct correlation with the antioxidant capacity was observed. Similar data were obtained in a study conducted with young, middle-aged and elderly participants using plums and grape juice [69,70]. Other studies demonstrate that many sleep-quality parameters, such as sleep efficiency, actual sleep time, total nocturnal activity, and immobility can be improved [71,72].

Also, healthy volunteers who drank one or two glasses of beer (330 or 660 mL) showed a clear increase in blood melatonin levels compared with basal melatonin levels 45 min after drinking, and an increase in blood antioxidant levels in the group that drank beer was observed. This effect can be related to the antioxidants in beer, such as organic acids, vitamin B, silicic acid, and flavonoids. One of the conclusions reached is that the phytomelatonin in beer may protect the body from oxidative stress damage due to its antioxidant properties [73]. In another cross-over trial, serum melatonin levels increased 2 h after tropical fruit consumption. Although the fruits consumed, namely orange, pineapple and banana have low phytomelatonin levels, this intake produced an up to 5-fold rise in serum melatonin levels [74,75]. This suggests that even fruit or plant extract containing low levels of phytomelatonin can influence blood melatonin levels.

The most relevant aspects related with human disease/disorders (see Figure 3) where the intake of phytomelatonin-rich plants might have some effects are listed.

### 3.2. Phytomelatonin as an Antioxidant

The excellent qualities of melatonin as an antioxidant are shown in the case of phytomelatonin. In this respect, it is interesting to note that melatonin shows certain particularities as an antioxidant: (1) the melatonin molecule has no pro-oxidative effects; (2) melatonin-intermediate products also show antioxidant properties; and (3) melatonin displays an important synergistic action with other antioxidants, such as ascorbic acid and glutathione, among others. Phytomelatonin obtained from natural plant extracts, i.e., without contamination or additions, can act together with other hydrophilic compounds, such as organic acids (vitamin C), phenolic acids, flavonoids, and anthocyanins. In some cases, it can also be accompanied by carotenoids (provitamin A) or tocopherols (vitamin E), which are compounds of a lipophilic nature. The presence of these compounds with phytomelatonin can enhance its antioxidant properties through synergistic actions [76,77,78,79]. Recent data clearly indicate that an increase in phytomelatonin levels in the blood is accompanied by an increase in serum antioxidant capacity [66,80,81]. The combination of phytomelatonin with various plant antioxidants has not been described as having harmful effects, such as those of the pro-oxidant activity found in many synthetic vitamin supplements. Related to the antioxidant activity of melatonin are multiple beneficial health effects for combating diseases and disorders due to oxidative stress and aging.

### 3.3. Phytomelatonin as Anti-Carcinogen

Melatonin can act as an anti-carcinogenic and antitumor agent. This effect has been studied in multiple cancers including breast, lung, liver, renal, pancreatic, colorectal, testicular, endometrial, cervico-vaginal, skin and brain cancers, as well as lymphoma. The action of melatonin on cancer cells has been related with its ability to reduce DNA damage, up-regulate antioxidative enzymes, change the expression of growth- and differentiation-related genes, and reduce some mitogenic signals and the metastatic capacity of tumor cells through the control of certain oncogenesis-related genes, among others [16,81,82,83,84,85]. A number of cancers have been associated with low melatonin levels or with deficiencies of melatonin-receptors in damaged tissues. Some therapies use melatonin to stop tumor proliferation [13,15,86,87,88].

### 3.4. Phytomelatonin as Gastrointestinal Tract Protector

Melatonin can be produced in human organs other than the pineal gland. This so-called “extra-pineal melatonin” is generated by many tissues, such as the gastrointestinal tract, retina, leukocytes, bone marrow, cerebral cortex, liver, thymus, spleen, heart, muscle, placenta, testis and skin [89,90,91,92]. In quantitative terms, extra-pineal melatonin is a 100 times more plentiful than that generated in the pineal gland, but it only seems to be released into the bloodstream for very short time periods [93,94]. However, it has been noted that large amounts of melatonin from the gastrointestinal tract enter the blood during the post-prandial response. Therefore, food intake results in an increase in plasma melatonin levels. These pulses of post-prandial melatonin seem to have little effect on the circadian clock, probably due to their brevity and the fact that they occur at the silent point (dead zone or zone where no phase shift occurs) in the phase response curve, which was experimentally used to describe the relationship between a stimulus (e.g., light pulses, melatonin dose) and a response during the circadian rhythm. Gastrointestinal melatonin prevents ulceration of the gastro-intestinal mucosa, reduces gastric hydrochloric acid secretion, stimulates the immune system and micro-circulation, and promotes epithelial regeneration, among other actions [93,94,95,96].

### 3.5. Phytomelatonin as Sleep-Quality Enhancer and Jet Lag Remedy

Another aspect of interest is that phytomelatonin-rich plants may be responsible for a sleep-inducing effect due to its known role in circadian rhythms, especially in sleep quality. Melatonin blood concentration is below 0.01 nanograms per mL during the day time and is about 0.2 nanograms per mL (maximum) at night [96]. Taking into account the phytomelatonin levels in some plants (see Table 1 and Table 2), an uptake of gastrointestinal phytomelatonin from plant foods might be expected. It has been suggested that the high levels of phytomelatonin in plants act in a compensatory manner in animals. The limited data that exist indicate that the consumption of plant foodstuffs containing phytomelatonin alters its circulating levels and increases 6-sulfatoxymelatonin (a catabolite) concentrations in the urine [37,97]. This may have implications with regard to its sleep-enhancing effect and certain pathological conditions associated with sleep, such as insomnia, among others. Indeed, age-related disturbances in the sleep–wake and temperature rhythms have been correlated with age-related reductions in the amplitude of the nocturnal melatonin peak [62,98,99]. In fact, the reduction of melatonin at an advanced age leads to disturbances of the circadian pacemaker, which causes internal temporal desynchronization in the central and peripheral clocks, inducing a variety of chronopathologies and leading to a generalized deterioration of health [11,63,100]. Thus, the consumption of plant foodstuffs could be beneficial as they may enhance the health effects mediated by phytomelatonin against sleep disorders. Also, the ingestion of phytomelatonin-rich foods could be involved in post-prandial sleepiness [5,62,99,101,102].

The intake of melatonin pills to attenuate jet lag is widespread [1,100]. The consumption of melatonin as a gero-protective agent seems to have many followers [11,103]. In this sense, it has been shown that oral administration of 1–300 mg melatonin or up to 1 g daily for 30 days had no adverse effects, although in some cases, somnolence and headache were described [104,105,106].

For patients suffering from sleep disturbances, it is generally recommended to take melatonin at different doses at the end of the afternoon or a few hours before bedtime. This often coincides with the end of an evening meal, providing an accumulative effect with post-prandial melatonin and the natural rise in pineal melatonin. All this means that in some patients, melatonin levels may be too high in the first few hours of sleep. Therefore, more studies are needed on the contribution of phytomelatonin in the bloodstream in the critical hours before bedtime. Given the wide variation in the pharmacokinetics of oral melatonin in humans, more studies are necessary to improve our knowledge of pharmacokinetics in healthy volunteers, in particular with respect to the effects of different doses, times of administration, and periods of treatment, as well as the relative contribution of dietary phytomelatonin.

## 4. Sources of Melatonin and Phytomelatonin

Structurally, melatonin and phytomelatonin are the same molecule. As mentioned above, “melatonin” refers to melatonin of synthetic or animal origin and “phytomelatonin” to that of plant origin. Practically all melatonin supplements that are marketed are made from synthetic melatonin, although some of plant origin can be found (see below). Previously, melatonin was obtained from animal sources such as cows, but due to the risk of viral infection, synthetic production is often preferred, using a simple and very productive process [107,108,109]. There are various production methods involving several synthetic routes, as shown Table 3.

Since the discovery of melatonin in 1958 by Lerner and collaborators [110,111], the organic synthesis of melatonin has been significantly improved with the arrival of more productive and economic processes. Routes 1 and 4 (see Table 3) appear to be the most feasible and commercially viable ones. Synthetic melatonin is generated in yields over 80%; a large number of side products, i.e., residual compounds of the melatonin preparation processes also appear. Table 4 shows some of the most common of these which are present in the commercially available synthetic melatonin preparations. Most occur at concentrations below 0.5%.

These contaminants can be classified according the synthetic route used [112,113]. Thus, in the “classical” organic melatonin synthesis from methoxyindoles (Routes 1, 2 and 3), the contaminants are related to tryptophan; these have also been described in tryptophan supplements, as shown in Table 4. Other contaminants, such as oxidized forms of melatonin or condensation-related products, arise from the instability of melatonin. In Route 4, several melatonin contaminants, derived from phthalimide, may appear. Up to 14 contaminants have been described in the organic synthesis of melatonin from phthalimide, some of which are listed in Table 4.

Eosinophilia-myalgia syndrome (EMS) is an incurable and sometimes fatal disease. In the mid-1990s, there were 27 deaths associated with EMS, but only a single case in 2011. It has been related to the presence of l-tryptophan derivatives in some poorly produced dietary supplements of tryptophan. These are mainly contaminants from the so-called “peak E” listed in Table 4. In the case of melatonin supplements, the risk of ingestion of these contaminants is much lower because the recommended daily dosage of melatonin is up to 1000 times lower than that recommended for l-tryptophan supplements [114,115]. With regard to phthalimide, this widely used chemical is currently subject to multiple toxicological investigations [116] but there are no conclusive data. However, the fact that phthalimide is present in toxic compounds such as pesticides and fungicides, suggests that some degree of toxicity is to be expected. Irritation of the eyes, dermatitis through skin contact, and respiratory tract sensitization are the only side effects that have been officially noted: “*Chronic Potential Health Effects: no information found at this time. The toxicological properties of this substance have not been fully investigated*” [116]. Some studies on possible teratogenic, mutagenic, and genotoxic effects due to similarities with thalidomide and fungicides (folpet, captan) have been suggested. In either case, there is a very small degree of risk involved in taking chemically synthesized melatonin supplements. In the case of phytomelatonin, its a priori natural origin should rule out any possibility of contamination due to chemical synthesis. Furthermore, phytomelatonin extracts are “accompanied” by a set of other substances including antioxidants, vitamins, simple phenols, flavonoids, carotenoids, and tocopherols, which are known to participate in the beneficial functions that phytomelatonin has in our body [57,117,118,119,120,121].

However, some risks still exist if phytomelatonin formulations have not been obtained properly. The presence of solvent residues due to extraction protocols is common. Also, the plant source used must be controlled to avoid the presence of pesticides or other compounds due to previous cultivation or postharvest treatments. The use of wild or organically grown plants as sources of phytomelatonin should avoid the presence of undesirable chemicals in supplements. For extraction, supercritical fluid extraction is recommended using carbon dioxide (CO_2_) at high pressure. CO_2_ is a good extraction solvent as it is pure, non-toxic, non-flammable, non-polar, stable, colorless, odorless and tasteless. Importantly, it is easily removed and highly selective. Industrially, carbon dioxide CO_2_ is used in beverages, foods, flavors and cosmetics, partially because of the value that is added to products processed in this way, since they can be labeled as environmental friendly. The application of “green chemistry principles” with the use of alternative solvents, ensuring minimal contamination, could perhaps be emphasized for this type of supplement [122].

In bioreactors, phytomelatonin is obtained using l-tryptophan as a precursor [48] (Figure 1). Such use leads to the situation described above, where a large amount of contaminants may appear in the formulations of “*apparently natural*” phytomelatonin. The only way to ensure the natural source of phytomelatonin supplements is through rigorous control of their origin (plant species and cultivation mode) and of the reagents and extraction processes used. The presence of “contaminants derived from l-tryptophan” can provide a clue with respect to the unnatural origin of phytomelatonin. The problem is that the identification of these contaminants requires sophisticated techniques, such as LC-MS or GC-MS.

## 5. Availability of Melatonin and Phytomelatonin Supplements

Nutraceuticals and dietary supplements are intended to enrich the diet. These are growing markets in both developed and developing countries. Some factors that make them interesting are their affordable prices, their availability as over-the-counter medication and the perception that they are natural and safe products. In the USA, the term “dietary supplements” is used, while the European Union (EU) uses the term “food supplements” in its regulations [123,124,125,126]. The EU includes them in the same regulations as those referring to foods, while the Food and Drug Administration (FDA) does not subject these products to control or evaluation, but requires that “*these statements have not been evaluated by the FDA*” appear on the label. Melatonin is categorized by the FDA as a dietary supplement, and the regulations applying to pharmaceuticals are not applicable to melatonin. In the USA, melatonin dosage is not limited, and melatonin supplements containing up to 10 mg can be found. In the EU, melatonin supplements contain less than 2 mg/unit; higher dosages are considered as drugs. 

Simple and composite formulations of synthetic melatonin come in a wide range of forms such as tablets, pills, sublingual drops, liquids, gels, creams, and even suppositories, and at dosages from 0.1 mg to 400 mg. In the case of composite formulations, synthetic melatonin is presented together with other compounds such as tryptophan, vitamins (C, B6), minerals, and even collagen and hyaluronic acid in the case of creams.

Lastly, some special preparations are available. These are formulations in the form of bi-layer tablets, with synthetic melatonin for fast onset of action combined with plant extracts for a slow action. The plants used in these formulations have relaxing, calmative, or sedative activities. Such plants include valerian (*Valeriana officinalis* L.), passionflower (*Passiflora incarnata* L.), scullcap (*Scutelaria galericulata* L./*S. lateriflora* L.), lemonbalm (*Melissa officinalis* L.), linden (*Tilia platyphyllos* L.), and Californian poppy (*Eschscholzia californica* Cham.). Aloe (*Aloe vera* L.), lavender (*Lavandula angustifolia* Miller), and avocado (*Persea americana* Miller) are used in creams.

At present, five commercial formulations exclusively composed of phytomelatonin are known. There is no evidence that there are experimental or clinical studies with these preparations rich in phytomelatonin. Even though the laboratories that market these formulations do not provide much information on their origin, it appears that in three cases synthetic melatonin is not used in these formulations. Some characteristics of these formulations with phytomelatonin are presented in Table 5.

In the case of Formulation #1, the information is scant. It is unknown whether the phytomelatonin is obtained entirely from rice plants, rice grains, or the waste products of the rice plant. The contents of phytomelatonin in rice plants are between 0.1 to 1.0 ng/g fresh weight (FW) [32]. Thus, taking into account the quantity of 1 ng/g of phytomelatonin in rice seeds, 300 kg of rice would need to be processed to obtain one capsule of 0.3 mg or 36,000 kg of rice per package. Surprising!

Formulation #2 (in Table 5) is the only one that indicates the source of phytomelatonin: freeze-dried Montmorency tart cherry skin extract and juice. The content of tart cherries is 14 ng of phytomelatonin/g fruit. To obtain one capsule of 15 micrograms, about 1 kg of cherries would be necessary and 64 kg of cherries per package. This appears credible.

Formulation #3 merely says that the phytomelatonin comes from a “*vegetarian source*”. As each tablet contains 10 mg of phytomelatonin, it is hard to calculate the tons of plant material needed to produce these capsules.

Formulation #4 is an anti-aging skin cream composed of a blend of selected botanical herbs that “*… contain full spectrum antioxidants … for healthier hair skin and nails*”, according to the label. In the information given, the company is committed to using phytomelatonin. The product contains the following plants: Chinese angelica (Danghui), *Lycium* fruit (goji berry), jojoba, scullcap, ginseng, Chinese licorice root (*Glycyrrhiza uralensis*), Chinese foxglove (*Rehmania chinensis*), and squisandra (*Schizandra chinensis*), among other ingredients (aloe, milk-vetch, cinnamon bark, manuka honey, etc.). Practically all the above herbs contain high levels of phytomelatonin, but since we do not know the relative proportions of each plant, the amount or concentration of phytomelatonin in this preparation cannot be calculated.

Formulation #5 is also a cosmetic cream. The manufacturer claims it is rich in phytomelatonin, but no information is provided regarding its content. Its label indicates that, in addition to other ingredients, the cream contains oils of almond, olive, oat, yarrow, salvia, jojoba and avocado, and botanical extracts of aloe, centifolia rose, sheabutter, and oregon-grape, and also some vegetable proteins.

Based on Table 1 and Table 2, the plant material used in Formulations 1–3 does not seem to have been optimally selected, since other sources contain much higher phytomelatonin levels. This is the case of many medicinal plants (Formulation #4 and #5). In many cases, the raw material is often a decisive factor in production costs. Therefore, plant by-products or waste from agricultural or food industries are often used. In the case of phytomelatonin supplements, on choosing low-cost raw materials, the levels of phytomelatonin in the plant material selected should be taken into account. Manufacturers should consider these facts.

A priori, using plants as a source of melatonin for therapeutic purposes would undoubtedly provide the assurance of avoiding multiple undesirable chemical by-products. However, at present there are no clinical or other tests that show the advantages of phytomelatonin compared with chemical melatonin. All this means that the research is in its initial stages; plant extracts rich in phytomelatonin and free of pesticides or other contaminants need to be obtained, so that clinical trials can be carried out to contrast the effects against those observed in studies made with chemical melatonin. Obtaining these extracts rich in phytomelatonin is now a priority for us.

## 6. Concluding Remarks

1. The presence of phytomelatonin in all plant species analyzed to date has opened the door to its use as a nutraceutical compound.
2. Aromatic and medicinal plants present higher phytomelatonin levels than ordinary vegetables. Such botanical herbs are optimal candidates for use in future melatonin supplements.
3. The search for and study of phytomelatonin-rich species and varieties should be regarded as a priority.
4. The control of growth conditions might help to obtain phytomelatonin-rich plants. A greater number of studies with respect to more plants and varieties is necessary.
5. Phytomelatonin has great potential as a therapy for many diseases both at physiological and pharmacological doses.
6. Its antioxidant, anti-carcinogenic, and rhythm-synchronizing properties represent an excellent area in which its use as a nutraceutical could have wide beneficial applications.
7. The absence of undesirable contaminants generated during the chemical synthesis of melatonin is the key factor in choosing phytomelatonin as a “100% natural” supplement.
8. New preparations containing phytomelatonin will inevitably appear, with synthetic supplements almost certainly being displaced by natural substances.
9. It is to be hoped that such products will contain natural substances whose origin and possible contamination will be strictly controlled.
10. However, the possible harmful effects of phytochemicals (other than phytomelatonin) of either vegetable origin (such as alkaloids), or of non-natural origin (such as pesticides), with a possible presence in plant extracts, should be taken into account and studied in depth.

## Figures and Tables

**Table 1:** Phytomelatonin content of some edible plant species.

Common Name/Species	Phytomelatonin Content (ng·g^−1^ DW or FW) ^a^	References
Coffee beans/*Coffea* sp.	5800–6500 DW	[34]
Goji berry/*Lycium barbarum* L.	530–103 DW	[35,36]
Kidney bean sprouts/*Phaseolus vulgaris* L.	529 DW	[37]
White radish/*Raphanus sativus* L.	485–0.6 DW	[20,35]
Jujube/*Ziziphus jujube* Lam.	256 DW	[35]
White mustard/*Brassica hirta* L.	189 DW	[36]
Apple/*Malus domestica* Borkh.	134–0.4 FW	[20,38]
Black mustard/*Brassica nigra* L.	129 DW	[36]
Sweet cherry/*Prunus avium* L.	120–8.0 FW	[39,40]
Tomato/*Solanum lycopersicum* L.	114–0.3 FW	[20,41]
Fenugreek/*Trigonella foecum-graecum* L.	43 DW	[36]
Bellpepper/*Capsicum annuum* L.	42–9.0 FW	[42]
Almond/*Prunus amygdalus* Batsch.	39 DW	[36]
Sunflower/*Helianthus annuus* L.	29 DW	[36]
Fennel/*Foeniculum vulgare* Mill.	28 DW	[36]
Grape/*Vitis vinifera* L.	18–1.2 FW	[43,44]
Alfalfa/*Medicago sativa* L.	16 DW	[36]
Cardamom/*Elettaria cardamomum* L.	15 DW	[36]
Strawberry/*Fragaria ananassa* Duch.	11.2–1.4 FW	[41]

^a^ DW, dry weight; FW, fresh weight.

**Table 2:** Phytomelatonin content of some aromatic/medicinal plant species.

Common Name/Species	Phytomelatonin Content (ng·g^−1^ DW)	References
Thyme/*Thymus vulgaris* L.	38,000	[43]
Chinese liquorice/*Glycyrrhiza uralensis* Fisch.	34,000	[45]
Sage/*Salvia officinalis* L.	29,000	[43]
St. John’s wort*/Hypericum perforatum* L.	23,000	[46]
Peppermint/*Mentha piperita*	19,500	[35]
Cat’s claw herb/*Uncaria rhynchophylla* Miq.	2460	[35]
Tokyo violet/*Viola philipica* Cav.	2360	[35]
Feverfew/*Tanacetum parthenium* L.	1700	[47]
Mulberry/*Morus alba* L.	1510	[35]
Aloe/*Aloe vera* L.	516	[35]
Clove/*Syzygium aromaticum* L.	446	[35]
Yarrow/*Achillea millefolium* L.	340	[48]
Figwort/*Scrophularia ningpoensis* Hemsl.	342	[35]
Korean mint/*Agastache rugosa* Kuntz.	300	[35]
Qin Jiao*/Gentiana macrophylla* Pall.	180	[35]
Scullcap/*Scutellaria amoena* C.H. Wright	178	[35]
Japanese honeysuckle/*Lonicea japonica* Thunb	140	[35]
Curcuma/*Curcuma aeruginosa* Roxb.	120	[35]

**Table 3:** Precursors in the different chemical synthesis routes of melatonin.

Chemical Synthesis Route	Initial Structure (Relevant Precursor)	Reference
1	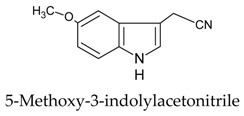	[107]
2	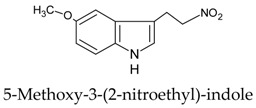	[107]
3	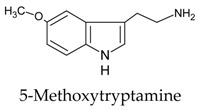	[107,108]
4	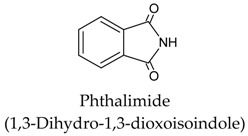	[108,109]

**Table 4:** Common contaminants in synthetic melatonin preparations.

Contaminant Compounds
1,2,3,4-tetrahydro-β-carboline-3-carboxylic acid
3-(phenylamino)alanine
1,1′-ethylidenebis-(tryptophan) (so-called peak E)
2-(3-indolylmethyl)-tryptophan
formaldehyde-melatonin
formaldehyde-melatonin condensation products
hydroxymelatonin isomers
5-hydroxy-tryptamine derivatives
5-methoxy-tryptamine derivatives
*N*-acetyl- and diacetyl-indole derivatives
1,3-diphthalimidopropane
hydroxy-bromo-propylphthalimide
chloropropylphthalimide

**Table 5:** Different commercial formulations containing phytomelatonin.

Formulation	Trademark	Origin	Dosis	Form (units)	Brand, Country
#1	HerbatoninPRO	Rice extract	0.3 mg	Capsules (120)	Natural Health Int., USA
#2	Sleep Support	Tart cherry skins Tart cherry juice	15 µg 3 µg	Capsules (60) Sachets (liquid)	Tru2U, New Zealand
#3	Melatonin 10 mg	Vegetarian	10 mg	Sublingual tablets (180)	Bioclinic Naturals, Canada
#4	Curaderm system	Diverse plants	-	Body Cream	Curapharm, USA
#5	Fitomelatonina	Diverse plants	-	Cosmetic Cream	Effegilab, Italy
